# Galactorrhea associated with juvenile systemic lupus erythematosus: a review of the role of prolactin

**DOI:** 10.1186/1546-0096-7-17

**Published:** 2009-10-23

**Authors:** Tova Ronis, Ciarán M Duffy, Karen N Watanabe Duffy

**Affiliations:** 1 Division of Rheumatology, The Montreal Children's Hospital, McGill University Health Center and McGill University, Montreal, Quebec, Canada

## Abstract

This case report is based on the clinical observation of a patient with juvenile systemic lupus erythematosus (SLE) who developed transient galactorrhea. The subsequent literature review documented an interesting association between prolactin and rheumatic diseases and in particular, hyperprolactinemia and SLE. The discussion that follows the case report explores this relationship and proposes a hypothesis regarding why this patient with juvenile SLE developed galactorrhea.

## Case report

A 13-year-old girl of Greek origin was initially referred to the Pediatric Rheumatology clinic at the Montreal Children's Hospital with Raynaud's phenomenon affecting her hands, feet, lips, and tongue. Her past medical history included intractable epilepsy since the age of two, secondary to cortical dysplasia, resulting in refractory complex partial seizures, despite a partial right frontal lobe resection and ongoing treatment with anti-convulsant medications. This situation was complicated by learning difficulties, behavioral problems, and developmental delay. Additional significant past medical history included migraines, precocious puberty and nocturnal enuresis. Also, she had been mildly anemic for two years and was found to be iron deficient. At the time of her initial assessment, her medications included two anti-convulsants, topiramate (sulfamate substituted monosaccharide) and lamotrigine (phenytriazine class). She had been taking risperidone 1.5 mg, an anti-psychotic agent (dopamine antagonist) that was being used during the school year to control aggressive behavior. Her mother had discontinued it, two weeks prior to her initial visit to the Rheumatology clinic, when the diagnosis of juvenile SLE was made.

Functional inquiry for systemic features of autoimmune disease was negative for rash, oral ulcers, hair loss, photosensitivity, gastrointestinal disturbances, skin tightening, muscle weakness, and urinary symptoms. She had begun menstruating six months prior to her presentation. Her menstrual periods were regular and lasted 7-9 days with heavy flow. The family history was significant for "mild" lupus in her mother.

Physical examination revealed a pleasant and cooperative adolescent whose weight and height were over the 95^th ^and 25^th ^percentiles respectively. The remainder of the exam was unremarkable.

Laboratory investigations revealed that her white blood cell count was mildly decreased (3.9 × 10^9^/L) with a decrease in absolute (1.3) and relative neutrophil counts. She was mildly anemic with a hemoglobin of 107 g/L; her platelet count was normal. Her erythrocyte sedimentation rate (ESR) was elevated at 43 (Winthrobe method) and her C-reactive protein (CRP) was negative. Urinalysis and renal function tests were normal. Antinuclear antibody (ANA) was positive (speckled at 1:160 dilution), extractable nuclear antigen (ENA) was positive for anti-Sm and RNP, anticardiolipin antibody (ACLA), as measured by ELISA (Inova), was positive in both the MPL and GPL fractions (twice the upper limit of normal) and she had a positive lupus anticoagulant (LAC). Her partial prothrombin time (PTT) was significantly elevated and she was Coombs' positive. Complement components C_3 _and C_4 _were initially normal but on subsequent evaluations, were mildly decreased. Her immunoglobulin levels were within the normal range. She had a significantly elevated anti-double-stranded DNA binding at 60%. Her thyroid function was normal and anti-histone antibodies were negative.

## What is the diagnosis and how would you manage this patient?

The clinical presentation of this patient was consistent with systemic lupus erythematosus (SLE). While she initially fulfilled three American College of Rheumatology (ACR) criteria for the classification of SLE (1. leukopenia, 2. a positive ANA, and 3. positive antibodies to Sm and the presence of antiphospholipid antibody (aPL)), she later developed a non-erosive arthritis thus satisfying the requirement for four criteria.

While lamotrigine-induced lupus has been described in the literature [1], our patient differed significantly, from a serological perspective. The patient with lamotrigine-induced lupus was anti-Ro/SSA positive and had normal anti-DNA, anti-phospholipid antibody and complement levels. In contrast, our patient was anti-Sm positive and was documented to have elevated anti-dsDNA binding, which is uncommon in drug-induced lupus. She also had mildly depressed complement levels at subsequent evaluations, in addition to positive anti-phospholipid antibody levels.

Similarly, while the induction of LAC by a combination of valproate and lamotrigine has been described [2], that patient's GPL was at the cutoff level considered to be positive and the MPL was normal. In contrast, our patient's GPL and MPL levels were significantly elevated to approximately twice the upper limit of normal. In addition, anti-histone antibodies were negative.

The patient met the serologic criteria for antiphospholipid antibody syndrome (APS). She was positive for both ACLA and LAC. While approximately 65% of children with SLE have aPL [3], she lacked the clinical manifestations of APS having never developed thrombosis and having never been pregnant. She was commenced on low-dose acetylsalicylic acid (ASA) because she was felt to be at risk for developing thrombosis given that she was documented to be LAC and ACLA positive. Treatment for APS in children is variable and has not been systematically studied [3]. A 2007 study investigated the use of prophylactic aspirin in asymptomatic APL positive adults and showed no benefit [4]. ASA is frequently used for prophylaxis of thrombosis in pediatric SLE patients, although the evidence in favor of this is lacking.

## Additional History

Two weeks later, the patient's mother revealed that the patient had experienced a milky, non-bloody discharge from both breasts for the preceding two weeks. There was no associated discomfort. This lasted approximately two weeks and stopped without any intervention. There was no history of trauma, unusual mental or physical stress, vigorous exercise or recent surgery. The patient was not sexually active and her beta-HCG was negative. Risperidone had been stopped one month before.

## What can cause galactorrhea?

Galactorrhea is most frequently caused by hyperprolactinemic states as seen in pregnancy, prolactinoma, primary hypothyroidism, drug-induced hyperprolactinemia (HPRL) and breast stimulation [5]. Other less common causes of HPRL include chest wall lesions, renal failure, hypothalamic-pituitary disease and tuberoinfundibular stalk disruption [5]. One third of women with HPRL experience galactorrhea [6] and most women with galactorrhea have HPRL [5].

## What is the relevance of galactorrhea in connective tissue disease?

Prolactin (PRL) is a versatile hormone with many functions including mammary growth, differentiation and lactogenesis. It is produced by the anterior pituitary gland and various extrapituitary sites including endothelial cells and immune cells. The inter-relationship between PRL and the immune system is well known [7]. PRL is known to regulate cellular function including proliferation, differentiation, angiogenesis, inflammation, and apoptosis and is implicated in lymphoproliferation, cytokine production and antibody secretion [7]. The human PRL gene is situated on chromosome 6, close to the major histocompatibility complex [7]. PRL is normally released in repeated pulses as a stress hormone. Levels may be increased by mental or physical stress, breast stimulation, vigorous exercise, trauma, surgery under general anesthesia, food ingestion and during sleep [8]. PRL secretion is under the control of hypothalamic releasing and inhibiting factors and is regulated via inhibition by dopamine (DA).

**Figure 1:**
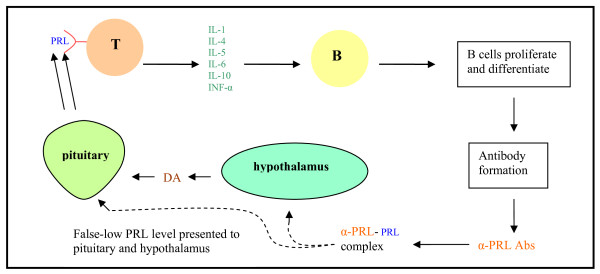
**Neuroendocrine immune interaction as proposed by Blanco-Favela et al**. [12]

Research has demonstrated that the endocrine and immune systems interact in a neuroendocrine immune loop. As such, rheumatic diseases can manifest endocrine components. Elevated PRL levels have been found to have proinflammatory effects [9] and activation of the prolactin receptor (PRL-R) has been shown to lead to the production and release of cytokines including IL-1, IL-4, IL-5, IL-6, IL-10 and INF-α [7]. This in turn, stimulates activated B cells to proliferate and differentiate supporting the hypothesis that PRL promotes a rupture of tolerance by stimulating increased immunoglobulin G (IgG) production [7]. (Fig. 1) The PRL-R has been identified in a number of cells and tissues including hematopoietic stem cells, T and B lymphocytes and almost all subtypes of immune cells and other important targets in SLE [10].

The first report of HPRL in SLE was in 1987 in male patients [10]. None of these patients had galactorrhea. Since then, HPRL (defined as serum PRL greater than 20 ng/ml for men, 25 ng/ml for women) has been reported in 15-31% of adult SLE patients. This is in contrast to the much lower frequency of HPRL in healthy women from 14 to 43 years of age of 3% [11]. There is contradictory data with regard to pre-pubertal SLE patients. Some studies report normal levels of sex hormones (PRL, follicle-stimulating hormone (FSH) and luteinizing hormone (LH), while others have identified from 9-31% of juvenile SLE patients with HPRL [12]. There has been no report of galactorrhea in any of the patients with primary HPRL and SLE.

HPRL in SLE may be explained by the stimulation of pituitary PRL secretion by cytokines. IL-1, IL-6 and other cytokines have been shown to stimulate release of PRL and other hormones from cultured rat pituitary cells. However, it is not well established whether cytokines in the peripheral blood affect neuroendocrine activity [10].

PRL secretion is inhibited by dopamine, and defects in DA metabolism have been described in patients with SLE. Lower levels of one of the main DA metabolites, homovanillic acid (HVA), have been documented in SLE patients as compared with controls. However, circulating levels of HVA do not reflect DA metabolism in the CNS [13]. When the DA antagonist metoclopramide was given *in vivo *to SLE patients, increased pituitary PRL responsiveness was demonstrated. This suggests that SLE patients have augmented functional dopaminergic tone and that they display more physiologic PRL inhibition than healthy controls [13].

Immune cells produce PRL under normal circumstances regulated by a tissue-specific mechanism, different from that controlling pituitary PRL synthesis [13]. T lymphocytes from patients with active SLE produce increased amounts of PRL as compared with controls [13]. Single nucleotide polymorphisms across the PRL extrapituitary promoter region have been associated with a relative overexpression of the PRL gene in SLE [14]. Research has shown that under unusual circumstances, such as in hypophysectomized animals, this extrapituitary PRL can be "exported" in significant amounts to the general circulation to fulfill biological functions [13]. Therefore, it is possible that the excess PRL seen in HPRL SLE patients is not coming from the pituitary, but rather, from the immune system.

Experimental evidence from animal models supports a role for PRL in autoimmune diseases. It has been proposed that HPRL may accelerate autoimmunity [12], may be associated with the development of SLE, and has been associated with clinical disease activity of SLE during pregnancy [10]. Clinical trials using bromocriptine to inhibit PRL secretion by the pituitary gland in SLE patients have demonstrated improvement in their clinical course [12] and flares in disease activity have been reported after its withdrawal [10]. HPRL in SLE has been correlated with increased disease activity as measured by SLE disease activity index (SLEDAI), with active neuropsychiatric lupus, active lupus nephritis, raised ESR, anemia, lymphopenia, thrombocytopenia, and hypocomplementemia. Also, it has been associated with enhanced synthesis of ANAs, anti-dsDNA, ACLAs, and responsiveness to therapy [10].

Anti-prolactin antibodies (α-PRL Abs) have been reported in approximately 5% of adult SLE patients [10] and 6% of pediatric SLE patients [12]. The major isotype of the α-PRL Abs was IgG [9]. Forty percent of SLE patients with idiopathic HPRL have been documented to have α-PRL Abs with significantly higher PRL levels than SLE patients without these antibodies [12]. Furthermore, 100% of SLE patients with α-PRL Abs have HPRL [11].

The factors which cause the production of α-PRL Abs in SLE are not well understood. The presence of α-PRL Abs has not been shown to correlate with disease activity as measured by the SLEDAI [11]. One study found that the PRL-IgG complex exerted complete biological action *in vitro *in samples from patients with idiopathic HPRL [15]. This was measured with the Nb2 lymphoma cell assay which is an in vitro assay on a specific cell line that specifically assesses the bioactivity of PRL. In contrast, the PRL-IgG complex may not exert much action *in vivo *as it does not readily cross the capillary walls because of its high molecular weight. This might explain why SLE patients with HPRL and α-PRL Abs do not usually develop clinical manifestations such as amenorrhea and galactorrhea [12].

α-PRL Abs may act in HPRL by inducing a secondary form of PRL. Two possible mechanisms have been suggested: First, free PRL is readily filtered by the glomerulus but the larger PRL-Ab complex could escape degradation in the kidney. A second mechanism that has been proposed is that the α-PRL Ab could block the autoregulatory feedback mechanisms, resulting in presentation of an apparently false low level of serum PRL to the hypothalamus and pituitary [11]. (See Fig. 1)

A 2001 study of a pediatric population with SLE found idiopathic HPRL in 31% of their participants [12]. This was comparable to the incidence found in adult SLE and as in adults, it was more common in females than in males. There was a statistically significant positive correlation between serum PRL levels and disease activity and HPRL and disease activity as measured by the SLEDAI. There was no correlation between α-PRL Abs and disease activity [12]. This data can be compared with older studies that found a 9% frequency of HPRL in pediatric SLE [16]. This discrepancy can be explained by differences in the patients' ages or in PRL measurement technique [12]. Pediatric HPRL SLE patients without α-PRL Abs have reported varying menstrual abnormalities including primary and secondary amenorrhea. SLE patients with autoantibodies against PRL did not report these abnormalities [12]. Most adult SLE patients with HPRL do not show expected abnormalities such as menstrual disturbances and galactorrhea [12].

## What caused this patient's galactorrhea?

Potential causes for galactorrhea in this patient were excluded such as pregnancy, prolactinoma (the MRI demonstrated a prominent pituitary gland, without any focal abnormalities), renal failure (normal renal function tests and urinalysis), hypothalamic-pituitary disease (normal gonadotropin levels), and primary hypothyroidism (normal T4, TSH). There were no abnormalities noted on the ophthalmologic examination of the visual fields.

Although the patient had taken risperidone regularly for 8-9 months, it had been discontinued one month prior to the onset of galactorrhea. Risperidone, a dopamine antagonist is known to induce HPRL, however, the mean half-life of risperidone and its active metabolite, 9-hydroxyrisperidone, is 20 hours with a range from 21 to 30 hours. Due to its short half-life and the fact that the medication had been discontinued approximately one month prior to developing galactorrhea, make it unlikely that risperidone was the cause. Furthermore, there is limited evidence from a study of healthy volunteers that demonstrated reduction of the total active moiety of risperidone, when administered in combination with topiramate, as in this case [17].

The patient's serum prolactin level (7.5 ug/ml) was within normal limits and was measured on a single sample at the time that the patient's galactorrhea was resolving. Due to the transient nature and spontaneous resolution of galactorrhea in this patient, further prolactin levels were not obtained at that time. Even so, such documentation may be difficult since prolactin is released in pulses and levels vary with circadian rhythms and the menstrual cycle so it is recommended that repeated samples be obtained in resting patients [8]. Furthermore, heterophilic antibodies from human serum can react with the immunoglobulins included in the assay thereby interfering with the *in vitro *immunoassay [18]. Thus, a single normal result does not rule out transient HPRL as the cause of this patient's galactorrhea.

In conclusion, there is a well-established relationship between PRL and the immune system and specifically, in patients with SLE. However, galactorrhea has not previously been reported in these patients. In this case, it was likely due to multifactorial influences in this patient. Her previous history of cortical dysplasia gives rise to the possibility that there are subtle structural and possibly functional abnormalities affecting the pituitary gland. Previous treatment with a dopamine antagonist may have sensitized an otherwise clinically quiescent state, within the context of a potentially hyperprolactinemic state, given the diagnosis of SLE.

## Consent

Written informed consent was obtained from the patient for publication of this case report and accompanying images. A copy of the written consent is available for review by the Editor-in-Chief of this journal.

## Competing interests

The authors declare that they have no competing interests.

## Authors' contributions

TR, CD, KWD participated in manuscript preparation. All authors read and approved the final manuscript.

## Authors' information

Tova Ronis, MDCM, was a medical student at McGill University and is currently a Pediatric Rheumatology Fellow at Lucille Packard Children's Hospital, Stanford University.

Karen N Watanabe Duffy, MD, FRCPC, Rheumatologist and Training Program Director, Division of Rheumatology, The Montreal Children's Hospital, and Assistant Professor, Department of Pediatrics, McGill University.

Ciarán M Duffy, MB, BCh, MSc, FRCPC, Director of the Division of Rheumatology and Associate Pediatrician-in-Chief, The Montreal Children's Hospital, and Professor, Department of Pediatrics, McGill University.

## References

[B1] Sarzi-Puttini P, Panni B, Cazzola M, Muzzupappa S, Turiel M (2000). Lamotrigine-induced lupus. Lupus.

[B2] Echaniz-Laguna A, Thiriaux A, Ruolt-Olivesi I, Marescaux C, Hirsch E (1999). Lupus Anticoagulant Induced by the Combination of Valproate and Lamotrigine. Epilepsia.

[B3] Lee T, von Scheven E, Sandborg C (2001). Systemic lupus erythematosus and antiphospholipid syndrome in children and adolescents. Current Opinion in Rheumatology.

[B4] Erkan D, Harrison MJ, Levy R, Peterson M, Petri M, Sammaritano L, Unalp-Arida A, Vilela V, Yazici Y, Lockshin MD (2007). Aspirin for primary thrombosis prevention in the antiphospholipid syndrome. Arthritis & Rheumatism.

[B5] Whitman-Elia GF, Windham NQ (2000). Galactorrhea may be clue to serious problems: Patients deserve a thorough workup. Postgraduate Medicine.

[B6] Yazigi RA, Quintero CH, Salameh WA (1997). Prolactin disorders. Fertility and Sterility.

[B7] Vera-Lastra O, Jara LJ, Espinoza LR (2002). Prolactin and autoimmunity. Autoimmunity Reviews.

[B8] Dostál C, Marek J, Moszkorzová L, Lacinová Z, Musilová L, Zvárová J (2002). Effects of stress of serum prolactin levels in patients with systemic lupus erythematosus. Annals of the New York Academy of Sciences.

[B9] Chikanza IC (1999). Neuroendocrine immune features of pediatric inflammatory rheumatic diseases. Annals of the New York Academy of Sciences.

[B10] Jara LJ, Vera-Lastra O, Miranda JM, Alcala M, Alvarez-Nemegyei J (2001). Prolactin in human systemic lupus erythematosus. Lupus.

[B11] Leaños-Miranda A, Pascoe-Lira D, Chávez-Rueda KA, Blanco-Favela F (2001). Antiprolactin autoantibodies in systemic lupus erythematosus: Frequency and correlation with prolactinemia and disease activity. The Journal of Rheumatology.

[B12] Blanco-Favela F, Quintal MaG, Chavez-Rueda AK, Leaños-Miranda A, Berron-Peres R, Baca-Ruiz V, Lavalle-Montalvo C (2001). Anti-prolactin antibodies in paediatric systemic lupus erythematosus patients. Lupus.

[B13] Méndez I, Alcocer-Varela J, Para A, Lava-Zavala A, de la Cruz DA, Alarcón-Segovia D, Larrea F (2004). Neuroendocrine dopaminergic regulation of prolactin release in systemic lupus erythematosus: A possible role of lymphocyte-derived prolactin. Lupus.

[B14] Vera-Lastra O, Mendez C, Jara LJ, Cisneros M, Medina G, Ariza R, Espinoza LR (2003). Correlation of prolactin serum concentrations with clinical activity and remission in patients with systemic lupus erythematosus: Effect of conventional treatment. The Journal of Rheumatology.

[B15] Hattori N, Inagaki C (1997). Anti-prolactin (PRL) autoantibodies cause asymptomatic hyperprolactinemia: bioassay and clearance studies of PRL-Immunoglobulin G Complex. Journal of Clinical Endocrinology and Metabolism.

[B16] El-Graf A, Salah S, Shaarawy M, Zaki S, Anwer S (1996). Prolactin hormone in juvenile systemic lupus erythematosus: A possible relationship to disease activity and CNS manifestation. The Journal of Rheumatology.

[B17] Canadian Pharmacists Association. CPS (2009). Compendium of Pharmaceuticals and Specialties, The Canadian Drug Reference for Health Professionals.

[B18] IMMULITE Prolactin product monograph. http://diagnostics.siemens.com.

